# Bioinformatics Analysis Discovers Microtubular Tubulin Beta 6 Class V (TUBB6) as a Potential Therapeutic Target in Glioblastoma

**DOI:** 10.3389/fgene.2020.566579

**Published:** 2020-09-18

**Authors:** Lan Jiang, Xiaolong Zhu, Hui Yang, Tianbing Chen, Kun Lv

**Affiliations:** ^1^Central Laboratory, Yijishan Hospital of Wannan Medical College, Wuhu, China; ^2^Key Laboratory of Non-coding RNA Transformation Research of Anhui Higher Education Institution, Yijishan Hospital of Wannan Medical College, Wuhu, China

**Keywords:** ANXA2, mitochondrial, glioblastoma, TUBB6, S100A11

## Abstract

Glioblastoma (GBM) has long been a major clinical research challenge to scientists. The pivotal role of the mitochondria related gene family in the promotion of GBM tumorigenesis is not clear. We detected that microtubular tubulin beta 6 class V (TUBB6) was one of 33 differentially expressed mitochondrial-focused genes (DEMFGs) in GBM, and considered that TUBB6 is a potential therapeutic target in GBM. TUBB6 was vital for GBM and marked as the key prognostic gene in primary GBM. Mutations of TUBB6 in GBM were rare. Only four TUBB6 co-expressed hub genes (ANXA2, S100A11, FLNA, and MSN) exhibited poorer overall survival rates in higher expression groups (*p*-value < 0.05). We have confirmed the up-regulation of TUBB6 and its partners, ANXA2 and S100A11 in GBM and validated their importance as prognostic factors in primary GBM. TUBB6 was significantly correlated with stromal score in GBM samples (*p*-value = 6.99E-04). This study aimed to assess the importance of novel hub genes by analyzing the expression, potential function and prognostic impact of TUBB6 in human primary GBM cancer.

## Introduction

Reliable biomarkers have been the subject of many classic studies in cancer research (Salamone et al., 2018). Pan-cancer research can play a pivotal role in the development of diagnosis, treatment plans and novel therapeutics (Gizem et al., 2020). Gliomas [ependymoma, astrocytoma, oligodendroglioma, brainstem glioma, and glioblastoma (GBM)] are malignant brain tumors. The median patient survival time for GBM patients is only 15 months. Glioblastoma has the highest incidence of all gliomas and is the most malignant (stage 4) on the World Health Organization’s (WHO) scale of severity (Zhao et al., 2020). Palliative treatments include surgery, radiotherapy and chemotherapy. In China, the annual incidence of GBM was 5–10 million new patients per year.

The discovery of potential biomarkers is important to improve diagnosis, prognosis, and targeted therapy of GBM. Recently, we detected the HMG-box family establishing the significance of SOX6 in the malignant progression of GBM (Jiang et al., 2020a), and found three core genes associated with survival in GBM (Jiang et al., 2020b). Besides, another study revealed that expression of 77 known genes can serve as biomarker in pan-cancer (Ji and Cui, 2020). In the last decade, the role of mitochondria in metabolic pathways and cell metabolism became apparent and may become a therapeutic target against cancer (Porporato et al., 2018), and play driver roles in some cancer types (Yuan et al., 2020) including GBM, such as the IDH1-mutated GBM cells that are full of mitochondria (Navis et al., 2013) and the importance of mitochondria in relation to altered energy metabolism (Khurshed et al., 2017). Mitochondria have become increasingly important in cancer research. Silencing of the mitochondrial protein VDAC1 (Voltage-Dependent Anion Channel 1) inhibits cell growth in GBM, lung cancer and breast cancer (Porporato et al., 2018). CHCHD2 (Coiled-Coil-Helix-Coiled-Coil-Helix Domain Containing 2) promotes malignancy and recurrence of GBM in the context of cell proliferation, metabolism, therapeutic resistance, and invasion (Lumibao et al., 2018). SIRT3 (Sirtuin 3) may well be a potential anti-GBM target for treatment via mitochondrial and PI3K/Akt pathways to induce GBM cell death (Wang et al., 2018). The connection of mitochondria and microtubules is also essential for independent segregation of mitochondria during mitosis (Chacko et al., 2019). Mitochondria provide ATP and are recruited by transportation along microtubules (Devine and Kittler, 2018). TUBB6 (Tubulin Beta 6 Class V) is a member of the beta tubulin superfamily, which is a major component of microtubules, and has a molecular weight of approximately 50 kDa. Microtubules contribute to many cellular processes, such as structural support, intracellular transport and DNA segregation (Findeisen et al., 2014).

**FIGURE 1:**
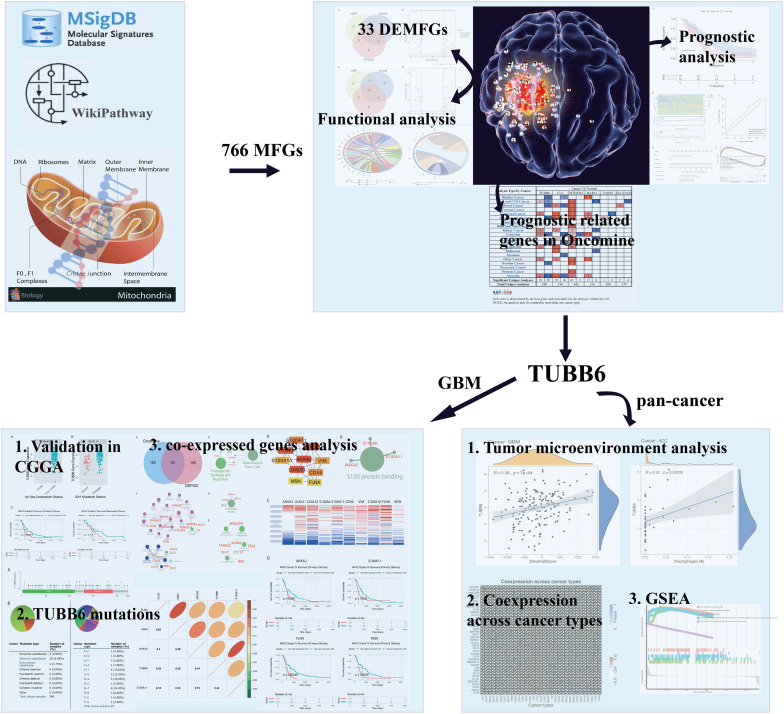
Flow chart of strategies in this study. The illustration of mitochondrion and GBM were downloaded from website: https://pulmonaryhypertensionnews.com/wp-content/uploads/2015/01/mitochondria.jpg and https://www.medicalnewstoday.com/articles/321809.

In this study, we hope to solve the following questions: (1) Which mitochondrial-focused genes are significantly differentially expressed in GBM? (2) Can we determine novel targets for anti-cancer treatment? (3) What are the potential functions of novel hub genes and co-expressed genes? In the present study, we applied a wide range of integrated bioinformatic approaches to assess the importance of these hub genes by analyzing the expression, potential function and prognostic impact of novel hub genes in human GBM cancer. The workflow diagram of all strategies is shown in Figure 1.

## Materials and Methods

### Dataset Collection and Functional Analysis

The gene normalized expression data and associated patient data were downloaded from the TCGA database for the GBM dataset (*n* = 174) (tumor: normal = 169:5) (Tomczak et al., 2015). We used the MSigDB v7.0 (Liberzon et al., 2011) and WikiPathway (Kutmon et al., 2016) in 2019 to detect the mitochondria-focused genes (MFGs). Differential expression analysis was performed using DESeq2 (Love et al., 2014), edgeR (Robinson et al., 2010) and limma (Smyth, 2005), and genes with adjusted *p*-value (*q*-value) <0.05 and fold change >4 were recognized as differentially expressed genes (DEGs), and Venn diagrams were drawn by VennDIS (Ignatchenko et al., 2015). Ggplot2 package was used to make a volcano plot.

The functional enrichment analysis of differentially expressed mitochondrial-focused genes (DEMFGs) was performed by R package “GOplot” (Walter et al., 2015), which visually displayed the GO annotations and KEGG pathway enrichment. The Multi-Protein Search module of DisNor was used to generate a protein interaction network linking DEMFGs (Lo Surdo et al., 2018), and visually displayed the first neighbor (level two). STRING v11.0 was used for protein interaction network analysis (Szklarczyk et al., 2018).

### Identification of Prognostic DEMFGs and Construction of the Risk Formula for Overall Survival Prediction

Univariate Cox proportional hazards regression was performed to obtain survival-related DEMFGs which were significantly connected to the overall survival (OS) of GBM patients in the training group (Zhao et al., 2018). After acquiring survival-related DEMFGs (*q*-value < 0.05), we excluded those that were not expressed in at least 10% of the samples. The remaining OS-related DEMFGs were then adjusted through the stepwise multivariate Cox regression model. Finally, those OS-related DEMFGs were selected for further analysis.

The subjects in each dataset were classified into a high-risk group and low-risk group according to the median risk score of the risk formula derived from the training set. This included 76 high-risk samples and 76 low-risk samples. To identify the potentially altered pathways in the high-risk group, we performed gene set enrichment analysis (GSEA) to scan Kyoto encyclopedia of genes and genomes (KEGG) pathways by “clusterProfiler” (Yu et al., 2012) in R. Explicitly, we constructed a pre-ranked gene list of all expressed genes ordered by log2FoldChange from the DESeq2 package in two groups. Significant pathways with *q*-value < 0.05 were identified.

The Oncomine database was selected to examine differences in mRNA expression of TUBB6 co-expressed key genes between GBM and normal tissues (Rhodes et al., 2007). The threshold limits were as follows: *p*-value < 1E-4; fold change >2; gene level, top 10%. For each key gene, we compared the results for GBM with those for normal tissues. GBM mRNA expression data was downloaded from CGGA data portal^1^, to further validate the key gene TUBB6. The prognostic value of TUBB6 expression for GBM patients was determined in CGGA. TUBB6 mutation analysis was produced by COSMIC database (Tate et al., 2019) and characterized in a pie chart. The frequency of TUBB6 mutations in GBM was tested by cBioPortal (Gao et al., 2018).

### Gene Association Analysis

GEPIA2 (top 199) (Tang et al., 2019) and Oncomine (correlation ≥ 0.50) (Rhodes et al., 2007) were used to retrieve TUBB6 co-expressed genes. GO and pathway enrichment analysis (*q*-value < 0.05) were performed by ClueGO (Bindea et al., 2009). Based on the co-expressed genes, GO analysis was performed in four categories: biological processes (BP), cellular components (CC), molecular functions (MF) (no significant enrichment results), and immune system processes (ISP); the pathway enrichment analysis included the Reactome, WikiPathway and KEGG pathway (no significant enrichment results).

Mutations of TUBB6 in GBM were analyzed using cBioPortal^2^ and COSMIC database (Tate et al., 2019). Forty co-expressed genes were co-expressed into a protein-protein interaction network by the STRING database (Szklarczyk et al., 2018), and the most important module was obtained by Cytoscape (CytoHubba plug-in) (Niissalo, 2007). Hierarchical clustering of the hub genes was performed by using the UCSC Cancer Genomics Browser. Gene correlation analysis was performed by “ggcorrplot” package in R.

### Tumor Environment Analysis in Pan-Cancer

Based on ESTIMATE database^3^, we downloaded estimate scores for each sample across all TCGA platforms. The files “estimate score” and “TUBB6 expression in pan-cancer” were uploaded into R to calculate the stromal and immune scores. CIBERSORTx (Newman et al., 2019) was a machine learning method which was used to establish the 22 immune cell subtype abundances from pan-cancer datasets (*p*-value < 0.0001). The correlation analysis between TUBB6 and cancer type and immune/stromal score/immune cell type was obtained using “ggplot2” package (*p* < 0.0001). The heatmap of TUBB6 co-expressed genes was designed by a “reshape2” package. Gene Set Enrichment Analysis analysis for TUBB6 in pan-cancer was analyzed by a “clusterProfiler” package (Yu et al., 2012).

## Results

### Identification and Functional Analysis of DEMFGs

**FIGURE 2:**
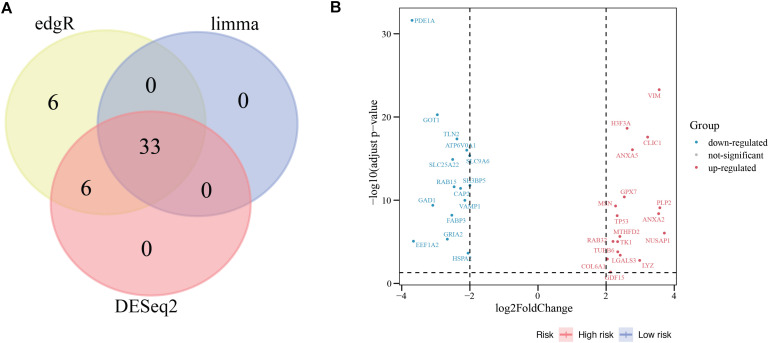
Differential gene expression. **(A)** Venn diagrammatic representation of DEGs by edgR, limma and DESeq2 (*q*-value < 0.05, logFoldChange >2). **(B)** Volcano plot of DEGs analysis.

We detected 766 MFGs in the MSigDB and WikiPathway databases, and 33 DEMFGs were identified by limma, DESeq2 and edgeR packages (Figure 2A), 18 DEMFGs were up-regulated and 15 DEMFGs were down-regulated according to their *q*-value < 0.05 and log_2_FoldChange >2 (Figure 2B).

**FIGURE 3:**
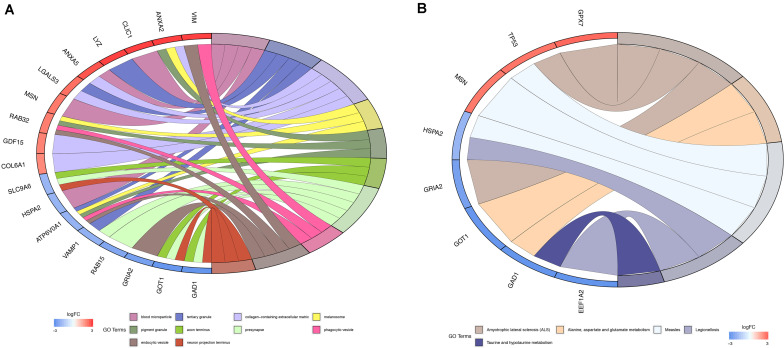
Function and pathway enrichment analysis. **(A)** Chord diagram for GO enrichment. **(B)** Chord diagram for KEGG pathway.

To determine the biological significance of DEMFGs, the chord diagram for GO terms and KEGG pathway were determined by GOplot (Figure 3). The top ten GO enrichments (*q*-value < 0.05) showed vesicles in the molecular functions of expressed transcripts during development, such as “blood microparticle”, “tertiary and pigment granule”, “axon terminus”, “endocytic vesicle”, and “neuron projection terminus.” The top one of the KEGG pathways was “amyotrophic lateral sclerosis (ALS).”

**FIGURE 4:**
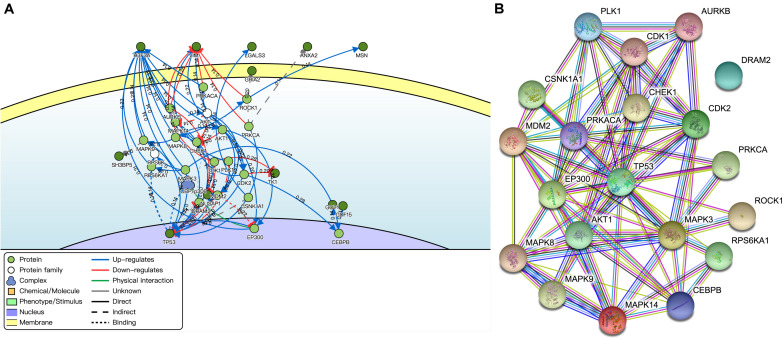
Interaction of DEMFGs. **(A)** The gene-gene interaction analysis by DisNor. **(B)** The protein-protein interaction analysis by STRING.

The key neighbor genes (first neighbors of twelve) were discovered by DisNor, among which five DEMFGs (H3F3A, VIM, LGALS3, ANXA2, and MSN) that are extracellular matrix genes, GRIA2 that is a cell membrane gene, another five DEMFGs (SH3BP5, TK1, NUSAP1, GDF15, and GOT1) that are cell matrix genes, and TP53 is a nucleus gene (Figure 4A). TP53 is activated by five key genes (CHEK1, 0.54; CDK2, 0.44; MAPK8, 0.42; MAPK14, 0.42; EP300, 0.56), and AURKB inhibits TP53 (score = 0.50). The AKT family induces MDM2 (score = 0.42) and AKT1 (score = 0.48), TP53 is indirectly activated by MDM2 (score = 0.59), and MDM2 inhibits TP53 (score = 0.83).

The protein-protein interaction (PPI) network was composed of an expected number of 47 edges and PPI enrichment *p*-value = 1.72E-05 by STRING (Figure 4B), which comprised 20 nodes and 78 edges (average node degree of 7.8 and average local clustering coefficient of 0.698).

### Prognostic Significance Analysis of DEMFGs

**FIGURE 5:**
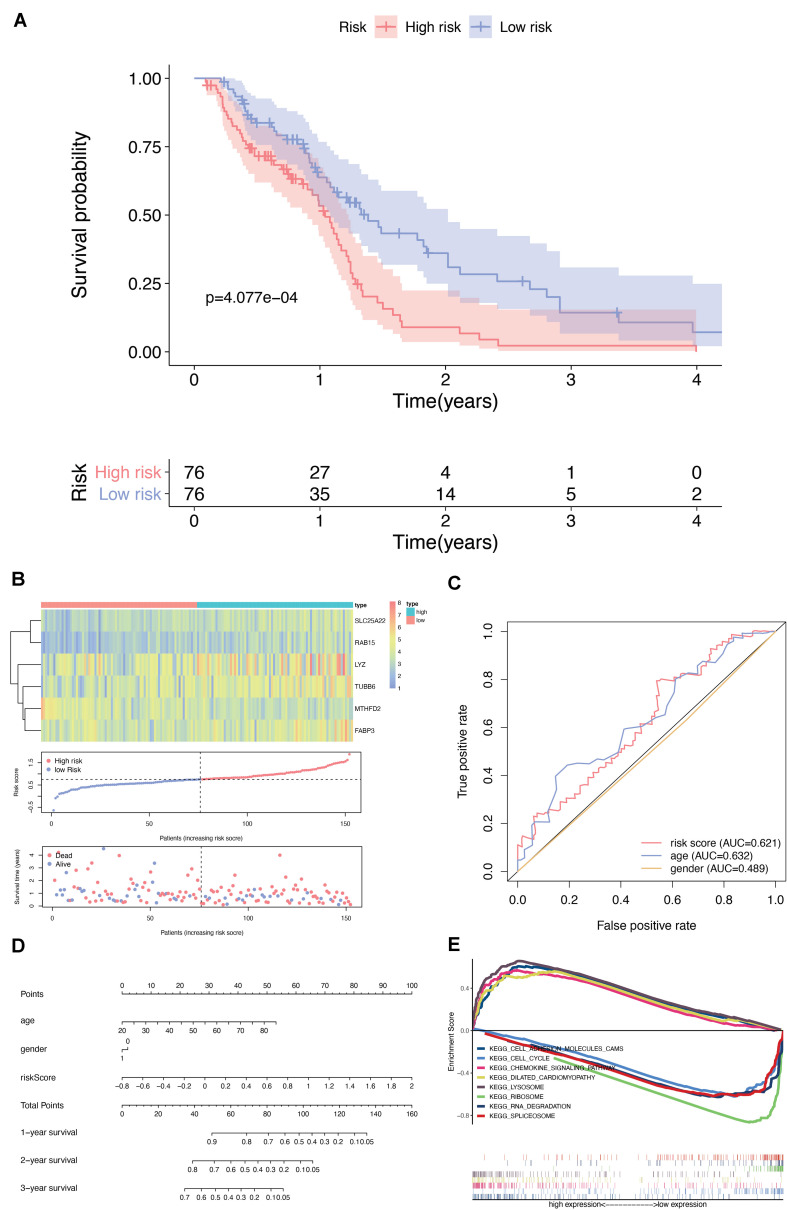
Screening and identification of DEMFGs. **(A)** Kaplan-Meier survival curves of the six-gene risk signature for TCGA-GBM dataset. **(B)** Heatmap of the six-DEMFGs in the high-risk and low-risk subgroups for the training set. The six-DEMFGs-based risk score distribution, patient survival status. **(C)** ROC risk six-gene risk signature distinguished the clinicopathological features of GBM. **(D)** Prognostic nomograms predicting the probability of 1-, 3-, and 5-years. **(E)** GSEA of KEGG pathways (MsigDB) significantly regulated in DEMFGs, *q*-value < 0.05 was chosen as cutoff for exploratory data analysis.

Univariate cox regression analysis of the DEMFGs was operated, and six genes (TUBB6, MTHFD2, LYZ, FABP3, SLC25A22, and RAB15) were marked as prognostic-related genes. We found significant differences between the high and low risk groups (*p*-value = 4.077E-4) via 5-years OS (Figure 5A). The distribution of six-DEMFGs for GBM patients was displayed by heatmap. The risk scores of patients in the training group were also ranked, and survival status (alive/dead) was plotted for each patient on a dot plot (Figure 5B). The area under the ROC curve (AUC) for the six-DEMFGs was 0.621, the age-dependent AUC (AUC = 0.632) indicated that the age score was a strong prognostic indicator for GBM patients (Figure 5C). The pathological stage, and age of these 6-DEMFGs were collected to build a nomogram with the aim of creating a quantitative method for the possibility prediction of OS at 1, 3, and 5 years for GBM patients (Figure 5D). We found that “cell adhesion molecules cams,” “cell cycle,” “chemokine signaling pathway,” “dilated cardiomyopathy,” “lysosome,” “ribosome,” “RNA degradation,” and “spliceosome” were significantly enriched in GSEA enrichment analysis (*q*-value < 0.05) to identify the potential pathways that differentiate the high-/low-risk groups, suggesting that these 6-DEMFGs may influence these pathways and thus influence the survival of GBM patients (Figure 5E).

### Validation of Prognostic-Related Genes in Oncomine and CGGA Databases

**FIGURE 6:**
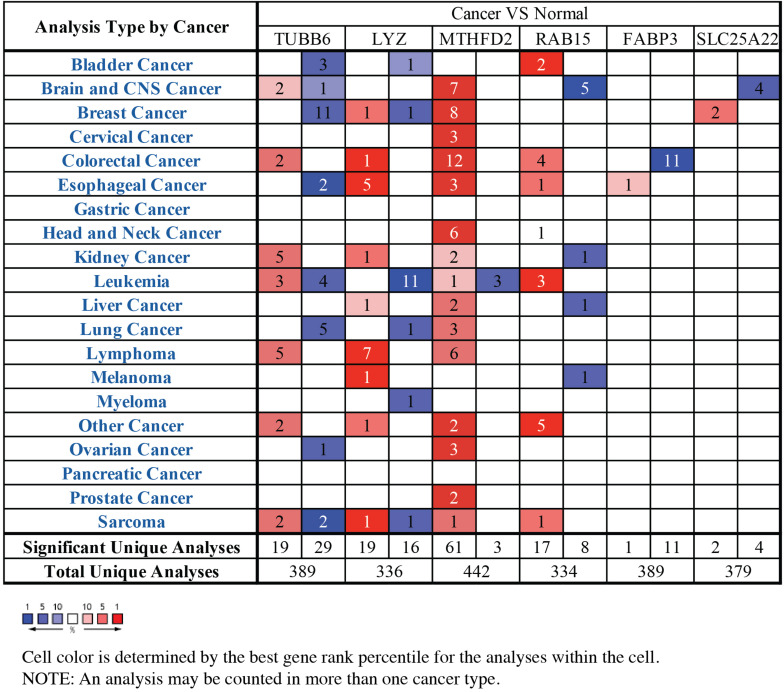
The mRNA expression patterns of prognostic-related genes in overall cancers. The mRNA expression difference between tumors and normal tissues were analyzed in Oncomine database. The number in the colored cell represents the number of analyses meeting these thresholds. The color depth was determined by the gene rank. The red cells indicated that the mRNA levels of target genes were higher in tumor tissues than in normal tissues, while blue cells indicated that the mRNA levels of target genes are lower in tumor tissues than in normal tissues.

Through the analysis of GBM vs normal tissues by Oncomine, we found that these prognostic-related genes were over-expressed not only in brain and CNS cancer but also in many other types of cancers (Figure 6). A total of 389, 336, 442, 334, 389, and 379 unique analyses for these six prognostic-related genes were found in the Oncomine database, respectively. There were one, five and four studies showing a statistically significant increase in the mRNA expression level of TUBB6, RAB5, and SLC25A22 in brain and CNS cancer tissues, in comparison with normal tissues. As for TUBB6 and MTHFD2, two and seven unique analyses of data with statistical significance revealed higher expression levels in cancer tissues than in normal tissues. This data suggested that the expression of TUBB6 and MTHFD2 was markedly higher in brain and CNS cancer samples than in normal tissues. Only TUBB6 was listed among the top 1% in GENE RANK of brain and CNS cancer, and we selected TUBB6 as the key prognostic gene for the following analysis.

**FIGURE 7:**
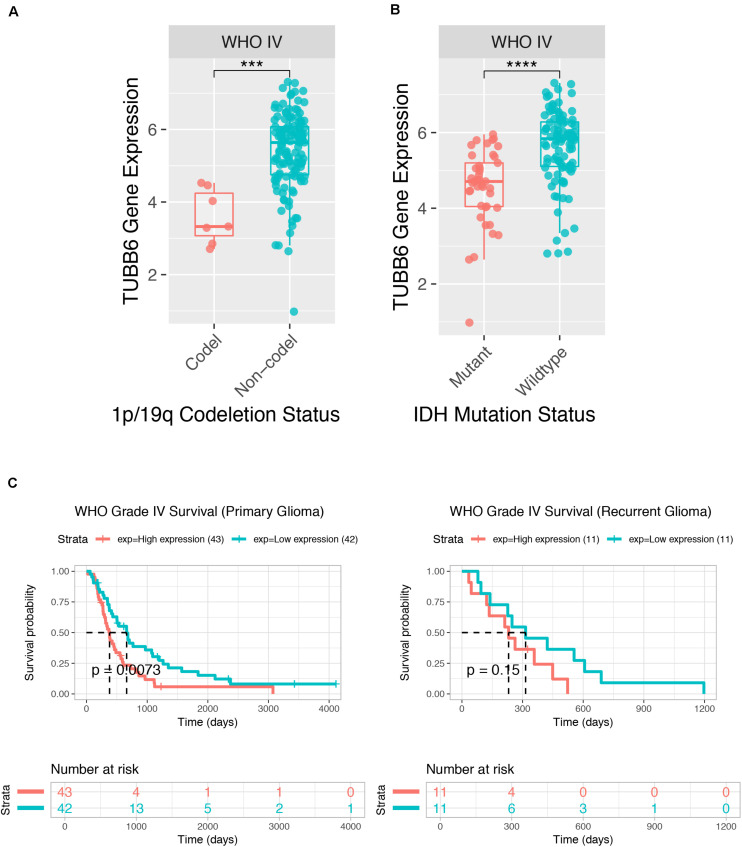
Prognostic value of TUBB6 for GBM patients in CGGA. TUBB6 expression in different types – codeletion status **(A)**, IDH mutant status **(B),** and survival probability **(C)**.

We further tested GBM patient data from CGGA, and the TUBB6 expression of codel/non-codel (Figure 7A), IDH-mutant/IDH-wildtype (Figure 7B) and the survival probability of primary GBM (Figure 7C) were found to show significant differences (*p*-value < 0.001), whereas, the survival probability of recurrent GBM was not significantly different, which shows that TUBB6 may be a reliable biomarker for primary GBM prognosis.

**FIGURE 8:**
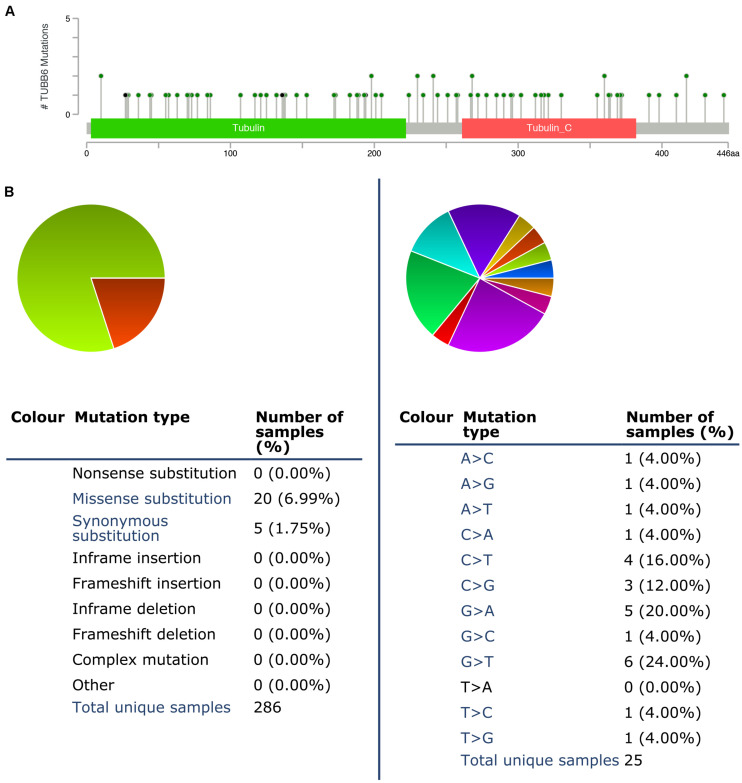
TUBB6 mutations in GBM. **(A)** Schematic representation of TUBB6 mutations (TCGA) using the cBioportal. **(B)** The percentages of mutation types of TUBB6 in GBM were indicated in a pie chart generated from COSMIC database.

We used cBioPortal to test the frequency of changes in TUBB6 mutations in GBM. The frequency of mutation was very low, only 0.01% (35182 unique samples, 286 unique samples with mutations) (Figure 8A). We analyzed the mutations of TUBB6 in GBM using the COSMIC database. The pie chart detailed the kind of mutations, including missense mutations and synonymous substitutions, the largest proportion of which were missense mutations, up to 6.99%. Nucleotide changes involved A > C, G, T; C > A, T, G; G > A, C, T, and T > A, C, G mutations, with the largest proportion being G > A and G > T (Figure 8B).

### TUBB6 Co-expression mRNAs in GBM

**FIGURE 9:**
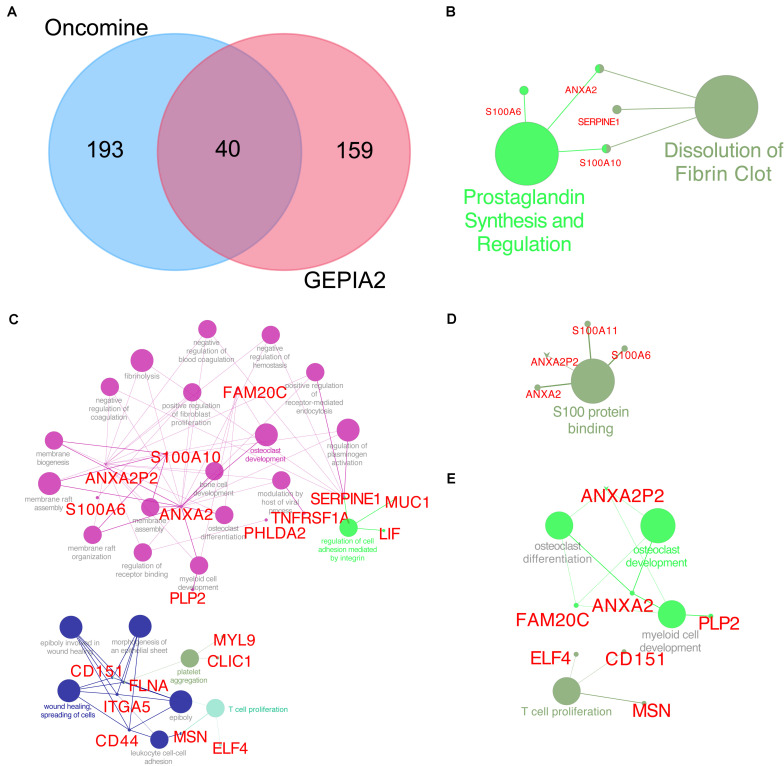
TUBB6 co-expression genes. **(A)** Venn plot for TUBB6 co-expressed genes between Oncomine (correlation >0.50) and GEPIA2 (top 199). **(B)** Reactome and wiki pathway of TUBB6 co-expression genes (the interact 40 genes). **(C)** Biological process, **(D)** cellular component, **(E)** immune system process of GO enrichment analysis (*q*-value < 0.05).

The Oncomine database was used to identify the top 233 co-expressed genes of TUBB6 (correlation ≥ 0.50), and the GEPIA2 database was applied to gain top 199 co-expressed genes for GBM. Forty common co-expressed genes were found in the two databases (Figure 9A). To analyze the biological characterization of co-expressed genes, we used the ClueGO method for functional enrichment analysis. The notable exceptions included prostaglandin synthesis regulation and dissolution of fibrin clots in pathway enrichment analysis (*q*-value < 0.05) (Figure 9B). GO enrichment (*q*-value) analysis showed that the biological processes including negative regulation of coagulation, membrane biogenesis, positive regulation of fibroblast proliferation were significantly affected (Figure 9C), consistent with S100 protein binding in the cellular component (Figure 9D), and osteoclast development and differentiation, T cell proliferation in immune system processes (Figure 9E). Collectively, these data indicated an essential role of TUBB6 in affecting cell development and proliferation in GBM.

### TUBB6 PPI Network Construction and Analysis of 10 Hub Genes

**FIGURE 10:**
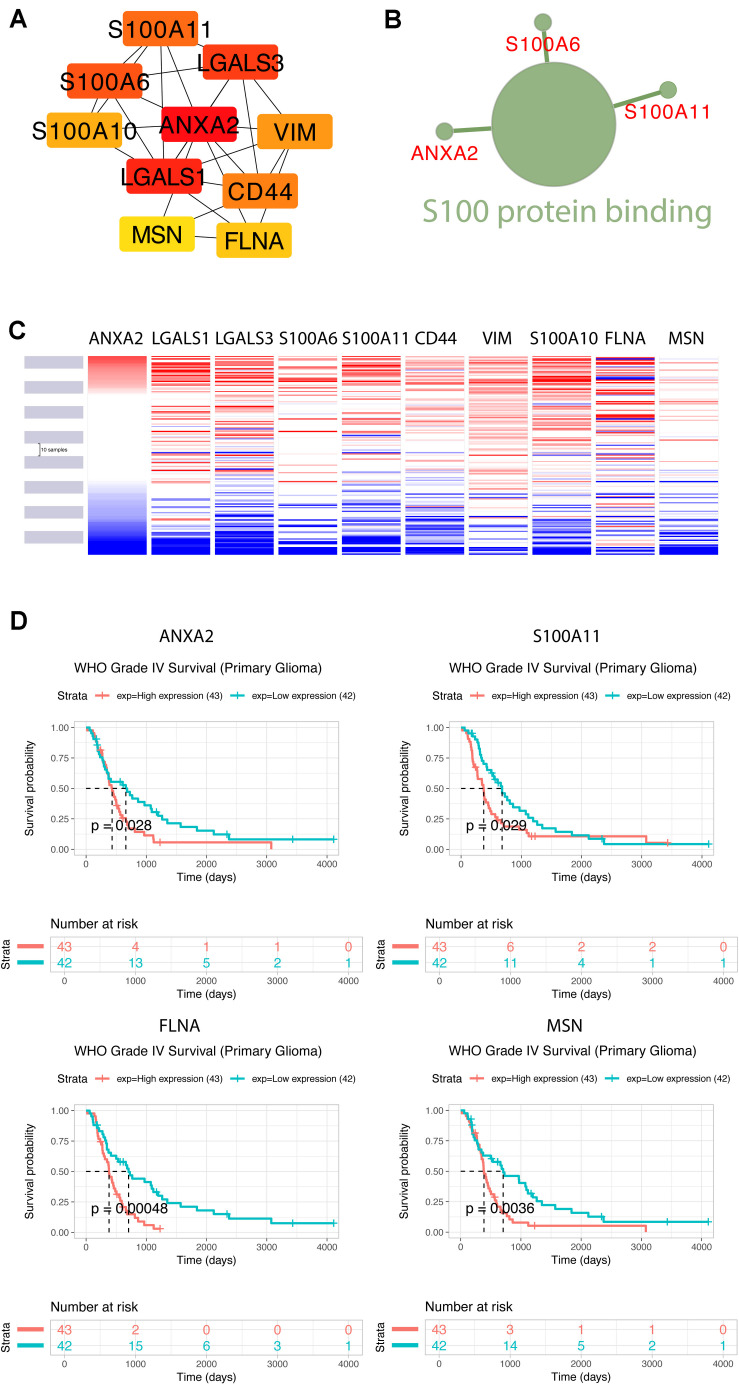
Construction of TUBB6 co-expression genes and analysis of hub genes. **(A)** The hub genes of TUBB6 co-expressed genes were characterized using cytoHubba. **(B)** The biological process analysis of hub genes was performed using ClueGO (*q*-value < 0.05). **(C)** The hierarchical clustering of hub genes was constructed using the UCSC online database. **(D)** Over survival analysis of hub genes in GBM which is derived on CGGA database (*p*-value < 0.05).

Using the STRING database, the 40 co-expressed genes were constructed into a protein-protein interaction network, and we extracted the most important module using Cytoscape (CytoHubba plug-in) (Figure 10A). The top ten genes included ANXA2, LGALS1, LGALS3, S100A6, S100A11, CD44, VIM, S100A10, FLNA, and MSN. The biological process analysis of hub genes was further performed by ClueGO plug-in. Particularly, S100 protein binding was altered, annexins and S100 proteins are two large but distinct calcium−binding protein families (Figure 10B). We performed hierarchical clustering of the hub genes using UCSC Cancer Genomics Browser (Figure 10C), detecting the concordant expression pattern across 10 genes. The OS of hub genes was analyzed using the Kaplan-Meier curve by the CGGA database (mRNA 325). Only four hub genes exhibited a poorer OS rate in higher expression groups (*p*-value < 0.05) (Figure 10D).

To identify the gene association and its co-associated genes with TUBB6, we performed a correlation analysis (Supplementary Figure S1). S100A11 and ANXA2 (correlation = 0.75) showed a positive correlation with TUBB6 (correlation > 0.40).

### The Importance of TUBB6 and Co-expressed Genes in Pan-Cancer

**FIGURE 11:**
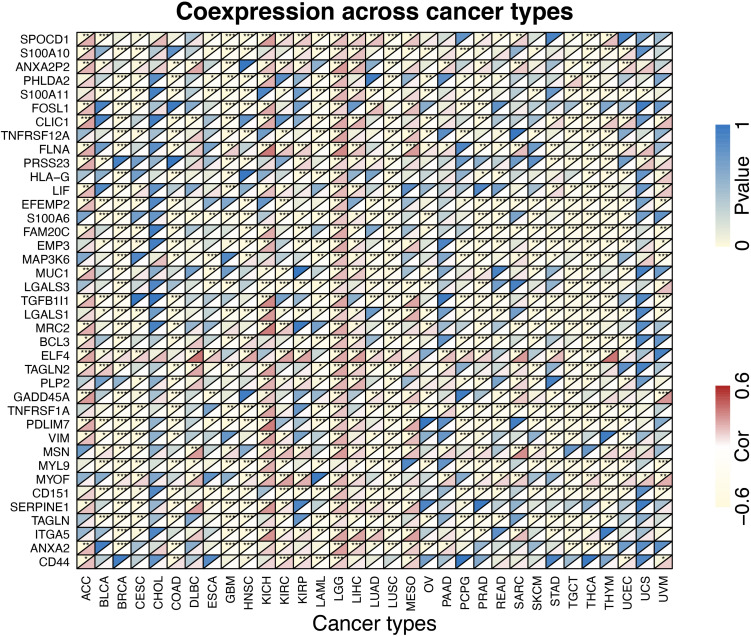
The heatmap of TUBB6 coexpression across cancer types.

The proportions of stromal score, immune score and immune-infiltrating cells in pan-cancers are shown in Supplementary Table S1. TUBB6 was significantly correlated with stromal score in 18 cancer types and with immune score in 17 cancer types (Supplementary Figure S2). Interestingly, TUBB6 was significantly correlated with the stromal score in GBM samples (*p*-value = 6.99E-04), whereas it was not significantly correlated with immune score. TUBB6 in ten cancer types was correlated with M0 macrophages, especially in LGG (*p*-value = 4.92E-06) (Supplementary Table S1). TUBB6 in HNSC (head and neck squamous cell carcinoma) was significantly correlated with ten immune-infiltrating cells, such as CD8 + T cells (*p*-value = 1.42E-07) (Supplementary Table S1). To better understand the differences in function, GSEA was used to evaluate TUBB6 in GBM (Supplementary Figure S3). TUBB6 was significantly enriched in the azurophil granule lumen and the B cell receptor signaling pathway. Next, the heatmap is visually analyzed the correlation of TUBB6 co-expressed genes in pan-cancer (Figure 11). DLBC (correlation = 0.61, *p*-value = 3.96E-06) and THYM (correlation = 0.61, *p*-value = 1.65E-13) were significantly positively correlated with ELF4. TGCT (correlation = −0.61, *p*-value = 5.85E-17) was significantly negatively correlated with EFEMP2.

## Discussion

GBM is an aggressive brain tumor with a need for deeper understanding and new therapeutic approaches in adults and children. The treatment of GBM is also a formidable challenge, which is correlated with poor patient prognosis (Meneceur et al., 2020). Despite the many in-depth studies of GBM treatment, however, the morbidity and mortality of GBM remain high (Zhao et al., 2020), and a better understanding of the tumor genetics of GBM is essential. Thus, finding key genes and understanding their function in controlling GBM development are pivotal to successfully curing GBM patients. This requires the development of suitable GBM biomarkers, which properly represent the aggressiveness of GBM, can be detected in the clinics and recapitulate the key characteristics of the disease.

Significant differences in gene expression associated with mitochondrial metabolism may show the potential involvement of mitochondria in GBM-treatment susceptibility. Previous studies showed that metabolic and mitochondrial genes were highly correlated with PGC1α in GBM cells (Wong et al., 2020). Mitochondrial metabolic inhibitors have been reported to activate a mitochondrion-to-nucleus stress signaling network that leads to alterations in gene expression, which affects a wide variety of cellular processes. Mitochondria are key organelles for cellular bioenergetics and constantly undergo dynamic remodeling processes, and increased production of reactive oxygen products is associated with a variety of human disorders (Goubert et al., 2017). Moreover, there is a lack of research on mitochondrial metabolism in GBM.

A sufficient supply of energy is essential for the proper function of the brain, and mitochondria have a pivotal role in preserving energy homeostasis (Goubert et al., 2017). Therefore, we linked potential mitochondrial metabolism genes to GBM. We detected 33 DEMFGs and compared them in GBM tissues and normal tissues. To better study the biological function, we performed GO enrichment and KEGG pathway analysis of these 33 DEMFGs. The biological processes of blood vessels were significantly enriched, such as “blood microparticle,” “endocytic vesicle,” and “neuron projection terminus”, which may well be related to tumor angiogenesis. According to the analysis of univariate cox regression, we evaluated the six genes (TUBB6, MTHFD2, LYZ, FABP3, SLC25A22, and RAB15) as prognostic-related genes. MTHFD2 can be targeted by miR-940 to inhibit glioma progression via inhibition of mitochondrial metabolism (Xu et al., 2019), and is highly expressed in GBM patients with a long survival time. LYZ is marked as the aging-related gene which is controlled by NLRP3 in glioma progression (Li and Liu, 2015). HIF1A induces FABP3 to facilitate FA uptake in GBM cell lines (Chen and Li, 2016). SLC25A22 is important as principal gate for glutamate homeostasis in astrocytes (Goubert et al., 2017). RAB15 interacts with FGFR1 involved in the recycling of glioma cell receptors and can be used as a pharmacological target to inhibit or down-regulate the proliferation of tumors by stimulating degradation (Giulietti et al., 2017). These results show that this model has considerable robustness in determining the prognosis of GBM patients.

The key prognostic gene TUBB6 (Tubulin Beta 6 Class V) is mapped on chr18:12,307,669-12,344,320 and is listed among the top 1% in GENE RANK of brain and CNS cancer. ROC curves of 1-, 3-, and 5-years survival rates of glioma patients show the accuracy of TUBB6 in the prognostic prediction of glioma patients. TUBB6 is used as a prognostic biomarker in many cancers, such as gastric cancer (Bai et al., 2020), ovarian cancer (Li et al., 2017), prostate cancer (Lin et al., 2019) and triple-negative breast cancer (Chung et al., 2017). However, the key role of TUBB6 in GBM has not been investigated yet. We validated TUBB6 with pathological features in GBM. As IDH mutation preceded 1*p*/19*q* codeletion, IDH1/2-mutant tumors are presented with or without 1*p*/19*q* codeletion (Arita et al., 2018). Primary GBM is the most frequently occurring GBM (90–95%) that occurs *de novo* without the IDH1mt, while secondary GBM has the IDH1mt (5–10%) and originates from glioma stage II and III (Molenaar et al., 2018). In this study, highly expressed TUBB6 with 1*p*/19*q* non-codeletion and IDH wild-type was found in GBM patients in the CGGA database. Interestingly, the expression of TUBB6 was higher in IDH wild-type GBM than IDH mutant GBM. The survival probability of primary GBM is significantly different (*p*-value < 0.001) compared to recurrent GBM. It is possible that in recurrent GBM patients TUBB6 is not a risk assessment indicator, which shows that TUBB6 may be a reliable biomarker for primary GBM prognosis.

We found 40 TUBB6 co-expressed genes in two databases. MSN may be the most favorable target in cell proliferation among these hub genes. ANXA2 plays key roles in the development of many malignancies and was shown in our study to be essential for GBM development (Tu et al., 2019). Humans and mice ANXA2 proteins are 97.6% identical. A previous study reported that S100A11 participates with ANXA2 to facilitate progression of GBM and to stabilize ANXA2 in GBM cells (Tu et al., 2019). ANXA2 is marked as core TUBB6 co-expressed gene in this study. On the basis of functional enrichment analysis, we found that ANXA2 was associated with regulating cell development, fibroblast proliferation and coagulation. ANXA2-S100A10 complex plays a key role in the progression of angiogenesis (Tantyo et al., 2019). ANXA2 and S100A11 may serve as prognostic markers in the validation of the CGGA database for survival in GBM. The correlation between TUBB6 and ANXA2/S100A11 was over 0.4, the higher correlation was S100A11-ANXA2 (correlation = 0.75). More studies are needed to identify the mechanism by which TUBB6 can be used as a therapeutic target in GBM and to find out how TUBB6 can affect the function of S100A11 and ANXA2 in GBM.

## Conclusion

In summary, we have confirmed the up-regulation of the expression of TUBB6 and its partners, ANXA2 and S100A11 in GBM and validated their importance as prognostic factors in primary GBM. We speculate that TUBB6 is a viable molecular target for the diagnosis and treatment of GBM.

## Data Availability Statement

All datasets presented in this study are included in the article/Supplementary Material.

## Author Contributions

LJ and KL: data curation and writing – review and editing. LJ, TC, HY, and XZ: formal analysis. HY, TC, and KL: funding acquisition. LJ: validation and writing – original draft. All authors contributed to the article and approved the submitted version.

## Conflict of Interest

The authors declare that the research was conducted in the absence of any commercial or financial relationships that could be construed as a potential conflict of interest.

## Footnotes

1 http://www.cgga.org.cn.portal.php

2 http://cbioportal.org/

3 https://bioinformatics.mdanderson.org/estimate/

## Supplementary Material

FIGURE S1 Corrplot represents of the expression of TUBB6 and hub genes.

FIGURE S2 Tumor microenvironment analysis for TUBB6 in pan-cancer.

FIGURE S3 GSEA plot for TUBB6 in GBM.

TABLE S1 The proportions of stromal score, immune score and immune-infiltrating cells in pan-cancers (*p*-value).
