# Supplementary figures and images for: Bioinformatics Analysis Discovers Microtubular Tubulin Beta 6 Class V (TUBB6) as a Potential Therapeutic Target in Glioblastoma
Source: Front Genet. 2020 Sep 18;11:566579. doi: 10.3389/fgene.2020.566579 (PMC7531581; doi:10.3389/fgene.2020.566579)

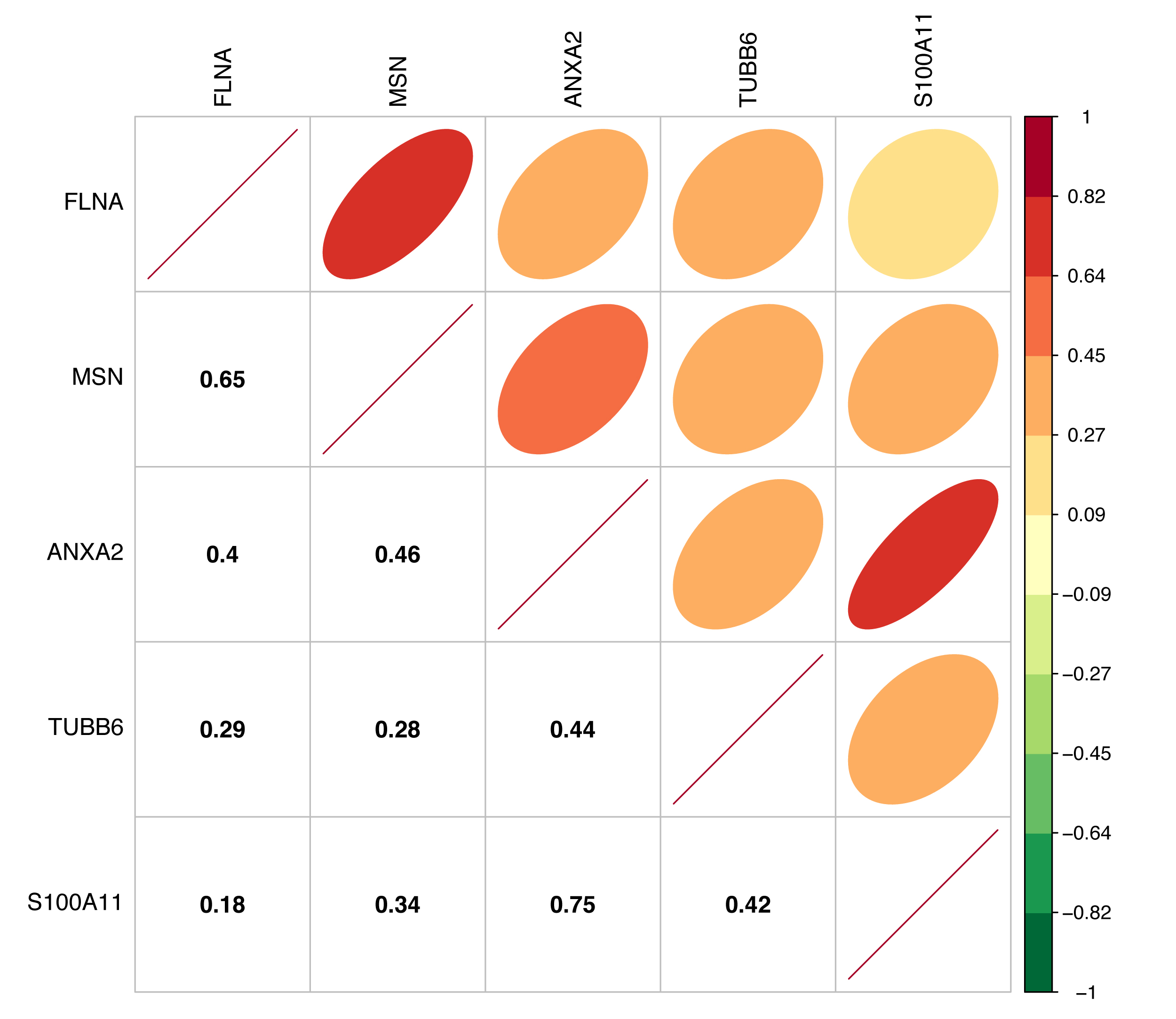

Supplement: FIGURE S1 — Corrplot represents of the expression of TUBB6 and hub genes. [file Image_1.JPEG]

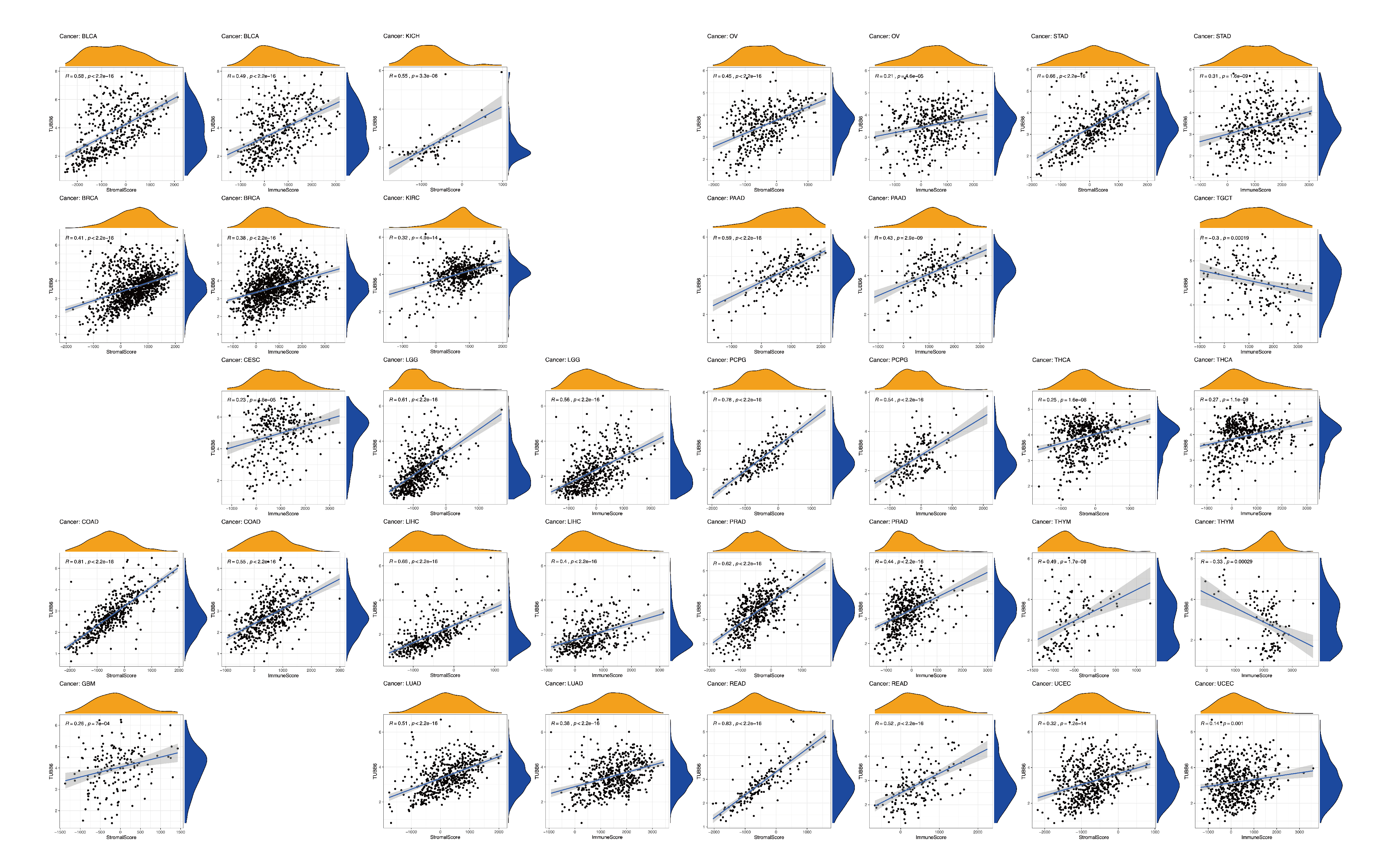

Supplement: FIGURE S2 — Tumor microenvironment analysis for TUBB6 in pan-cancer. [file Image_2.JPEG]

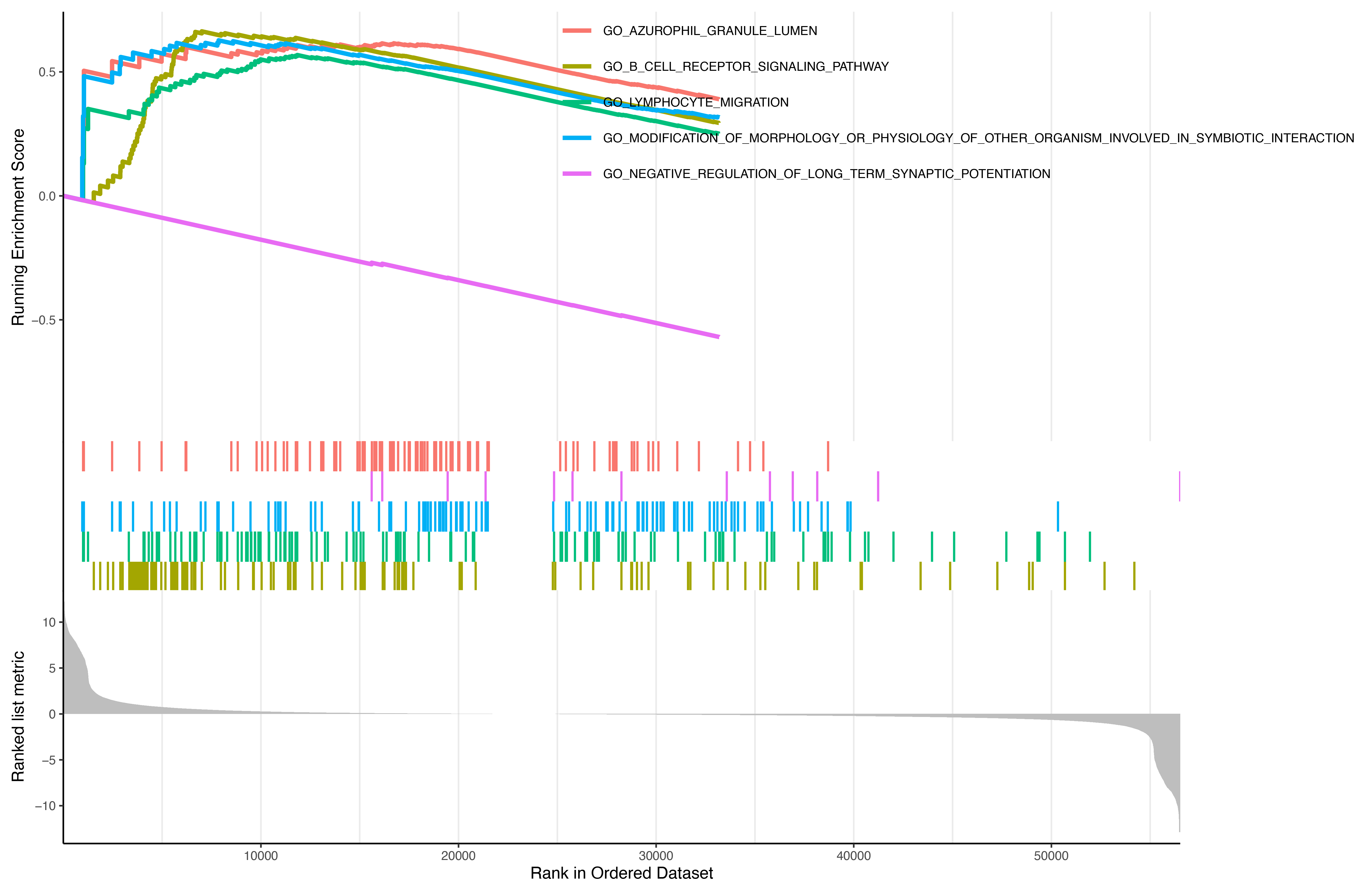

Supplement: FIGURE S3 — GSEA plot for TUBB6 in GBM. [file Image_3.JPEG]
